# Emission Characteristics and Factors of Selected Odorous Compounds at a Wastewater Treatment Plant

**DOI:** 10.3390/s90100311

**Published:** 2009-01-08

**Authors:** Eui-Chan Jeon, Hyun-Keun Son, Jae-Hwan Sa

**Affiliations:** 1 Department of Earth & Environmental Sciences, Sejong University, 98 Gunja-Dong, Gwangjin-Gu, Seoul, 143-747, South Korea; E-Mails: ecjeon@sejong.ac.kr; goodmrsa@empal.com; 2 Department of Environment and Health, Kosin University, 149-1, Dong Sam Dong, Young Do Gu, Busan, 606-701, South Korea

**Keywords:** Odorous compounds, emission characteristics, Dynamic Flux Chamber (DFC)

## Abstract

This study was initiated to explore the emission characteristics of Reduced Sulfur Compounds (RSCs: hydrogen sulfide, methyl mercaptan, dimethyl sulfide, dimethyl disulfide), ammonia and trimethylamine from a Wastewater Treatment Plant (WWTP) located at Sun-Cheon, Chonlanam-Do in South Korea. The study also evaluates flux profiles of the six selected odorous compounds and their flux rates (μg/m^2^/min) and compares their emission characteristics. A Dynamic Flux Chamber DFC was used to measure fluxes of pollutants from the treatment plant. Quality control of odor samples using a non-reactive sulfur dioxide gas determined the time taken for DFC concentration to reach equilibrium. The reduced sulfur compounds were analyzed by interfacing gas chromatography with a Pulsed Flame Photometric Detector (PFPD). Air samples were collected in the morning and afternoon on one day during summer (August) and two days in winter (December and January). Their emission rates were determined and it was observed that during summer relatively higher amounts of the selected odorous compounds were emitted compared to winter. Air samples from primary settling basin, aeration basin, and final settling basin were tested and the total amount of selected odorous compounds emitted per wastewater ton was found to be 1344 μg/m^3^ from the selected treatment processes. It was also observed that, in this study, the dominant odor intensity contribution was caused by dimethyl disulfide (69.1%).

## 1. Introduction

Odors are sensations resulting from the reception of a stimulus by the olfactory sensory system [1]. Humans are sensitive to a variety of odorous chemical compounds. The intensity, detectability, concentration and character of the chemical influence the human perception of an odor [2].

Most odor-producing substances found in domestic wastewater result from the anaerobic decomposition of organic matter containing sulfur and nitrogen. Inorganic gases produced from domestic wastewater decomposition commonly include hydrogen sulfide, ammonia, carbon dioxide and methane. Of these gases, only hydrogen sulfide and ammonia are malodorous. Often odor-producing substances include organic vapors such as indoles, skatoles, mercaptans and nitrogen-bearing organics [3].

Analytical and olfactometric approaches are the two ways that are used to measure odors. Characterization via chemical analysis as sensort or olfactometric characterization have advantages and drawbacks [4]. Complex mixtures, such as environmental air samples, contain many odorous compounds, generally at very low concentrations [5-8]. Analytical methods can identify each odorous compound from a complex mixture of odorants. With this method the concentration of each odorous compound can also be measured. Based on the characteristics of a certain type of odorous compounds, the sensitivity of the analytical method can even exceed the sensitivity of the human sense of smell.

Obnoxious odors from Wastewater Treatment Plants (WWTPs) have been of concern for many years. Recently there has been a greater social focus on odor related problems due to strict air quality regulations and increasing public concern with health and environmental deterioration [9]. Generally, odor emissions from WWTPs are from both point and area sources and are characterized by low concentrations and high air volumes over large areas. To determine the odor emission rate, knowledge of the flow rate and corresponding odor concentration are required. Usually large open area sources are significant contributors to overall odor emissions at WWTPs [10]. When measuring emissions from area sources, an enclosure device (flux chamber) is commonly employed to sample gaseous emissions from a defined surface area of the source. This involves determining the concentration of volatile compounds under a special cover in which aerodynamics and flow rates are controlled. The emission rate is expressed as the product of this concentration and flow rate.

Various types of reduced sulfur and nitrogen compounds behave as the key components of odor (and nuisance) [2, 9, 11]. Therefore, a precise description of the gas composition from Wastewater Treatment Plants (WWTPs) can be highly valuable in assessing the environmental impact of malodor issues in both the WWTPs and its surrounding areas [12-14]. This study has been initiated to explore the emission characteristics of Reduced Sulfur Compounds - hydrogen sulfide (H_2_S), methyl mercaptan (CH_3_SH), dimethyl sulfide ((CH_3_)_2_S), dimethyl disulfide ((CH_3_)_2_S_2_) - ammonia (NH_3_), and trimethyl amine ((CH_3_)_3_N) from a typical medium-sized Wastewater Treatment Plant (WWTP) in Korea. Table 1 presents the selected odorous compounds and their corresponding odor threshold values associated with domestic wastewater.

**Figure 1.**
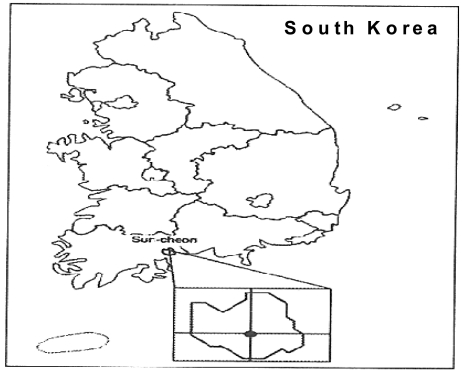
Location of Sun-Cheon Wastewater Treatment Plant.

The odor threshold refers to the minimum concentration required for an individual to perceive the odor, although the exact type of odor may not be identifiable [2]. A Wastewater Treatment Plant (WWTP), located at Sun-Cheon, Chonlanam-Do was chosen as the test facility (Figure 1). It was chosen as it represents a typical medium sized WWTPs in Korea. It employs the activated sludge treatment process, which is the most common treatment process for the Korean wastewater treatment plant.

In this study, emission characteristics of six selected odorous compounds from a WWTPs were investigated. Also, this study evaluated flux profiles of the six selected odorous compounds emitted from the water surface of the WWTP using a Dynamic Flux Chamber (DFC) which is found to be a suitable sampling device for area sources such as wastewater treatment plants. The paper provides various odorous compounds flux rates (μg /m^2^/min) based on the treatment processes at the WWTP. The results of this paper can be used as a background for possible contribution to the national and international study on emission characteristics and factors at WWTPs. Comparisons of odorous compounds emission characteristics based on various factors are also made.

## 2. Materials and Methods

### 2.1. Sun-Cheon Wastewater Treatment Plant as a Sampling Site

The emission characteristics of Reduced Sulfur Compounds (hydrogen sulfide, methyl mercaptan, dimethyl sulfide, dimethyl disulfide) and ammonia and trimethylamine were investigated as the major odorous compounds from WWTPs. As mentioned previously, a WWTP located at Sun-Cheon, Chonlanam-Do was chosen as the test facility. Primary settling basin, aeration basin, and final settling basin were selected as sampling sites for odor compounds at the WWTP. The test WWTP treats 130,000 tons of wastewater per day. Air samples were collected in the morning and afternoon on one day during summer (August) and two days in winter (December and January). Three days used to gather the samples will only give a rough estimate of the results. More samples will produce more accurate results taking into consideration the different weather conditions that may arise. Data was gathered in the three days because of the restrictions at that time. We were allowed to get samples only thrice.

The ambient air and sewage temperature during the summer season fell between 29.5∼32.4, (Average 31.3 °C) and 22.0∼24.2 (average 24.2 °C).The Winter's average temperatures were 9.6 °C and 12.4 °C for both the ambient air and sewage respectively. Table 2 shows the temperature and pressure of ambient air, DFC, and sewage surface during sampling.

### 2.2. Manufacturing the Dynamic Flux Chamber (DFC) for sample collection

**Figure 2.**
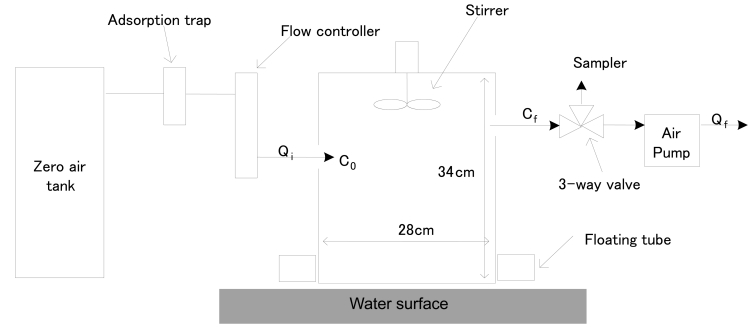
A Schematic Diagram of Dynamic Flux Chamber (DFC).

The DFC method can be used to measure pollutant fluxes from land or liquid surfaces. In the former case, the chamber is installed directly on the land surface, while a floating tube is inserted into the bottom of the chamber for the latter case [18-19]. As we intended to measure fluxes from a sewage treatment plant, a DFC system with floating tube was used to measure all flux values. Figure 2 shows a schematic diagram of the DFC that was used.

It was built with an acrylic wall and a dome shape on the top side. The wall of the acrylic chamber was covered with a polytetrafluoroethylene (PTFE) film to reduce sampling artifacts (e.g., reactions between the inner wall and odorous materials). The DFC system was operated by supplying clean air into the chamber inlet to estimate the flux. The flow rates of air entering and exiting the chamber were set to be only slightly different at 5 and 3 L/min, respectively. In order to maintain constant air flow in the DFC, a Teflon stirrer was operated at fixed rotating rates at all times. A vent hole was made on the top of the DFC to balance the pressure difference between the inlet and outlet of the chamber. A K-type thermocouple was also inserted through the top of the DFC to monitor temperature changes inside the chamber. A decompression union (made of a stainless steel material with a 1/4″ bulkhead union [Swagelok, USA]) was installed to maintain the inner pressure of the DFC similar to air pressure. All connection lines of the DFC system were made of 1/4″ Teflon tubing.

### 2.3. Quality control for odor samples with DFC

**Figure 3.**
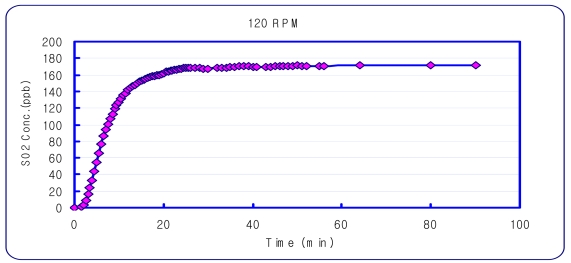
Concentration Variation inside the DFC with a 120 rpm stirrer.

An experiment was performed to determine the DFC concentration equilibrium time. Sulfur dioxide, which is a non-reactive gas, was used for this experiment. A Teledyne/API-100A SO_2_ Analyzer (USA), was used to measure sulfur dioxide. The amount of gas for the DFC inlet and outlet was set at 5 L/min and 3 L/min, based on previous research [19]. It was found that the most stable sampling condition was with a DFC stirring speed of 120 rpm, and sampling 60 minutes after setting the chamber (Figure 3).

### 2.4. Collection of odor samples

A lung sampling method was devised by building up an internal vacuum. This allows collection of an air sample without contacting the vacuum pump line. The lung sampler can be used to reduce possible sources of sample contamination. This sampling system was useful for collecting samples of sulfur compounds and trimethylamine. Initially, an empty Tedlar bag (5 or 10 L) was placed inside the lung sampler and connected to the sample inlet port. Then a vacuum was created inside the lung sampler by a vacuum pump. The valve was opened to pull an air sample stream into the Tedlar sampling bag. This vacuum sampling was operated to pull at a flow rate of 3 L/min measured at the DFC outflow. Cleaning of Tedlar bags involved flushing them with nitrogen gas for a period of about twenty-four hours. All Tedlar bags used for sampling were pre-conditioned more than once by the same sample gas prior to the actual sampling. Strongly absorbent odors can be partially absorbed on the inside wall of the DFC or sampling tube, or can react with other odorous compounds. Accordingly, the inside wall of the DFC was painted with Teflon to minimize ammonia sample loss.

### 2.5. Analysis of reduced sulfur compounds

To measure Reduced Sulfur Compounds (RSCs), a gas chromatography (GC, Model DS 6200, Donam Instruments, Korea) was interfaced with a pulsed flame photometric detector (PFPD, Model 5380, O.I. Co.) using a loop injection system. A thermal desorption unit (TDU, UNITY, Markes, Ltd., UK) could concentrate the gas samples using a cold trap and then transfer it to GC/PFPD system. To determine the optimum resolution between different RSCs, we used a DB-VRX column (60m × 0.32, 1.8 mm ID) with each cycle running at 20 min intervals. The sample volume was modified at each analysis depending on the sulfur contents of samples. The GC conditions for gas detecting system were set as shown in table 3.

A primary standard contained in a cylinder containing equimolar concentrations (10 ppm with 5% accuracy) of Reduced Sulfur Compounds (H_2_S, CH_3_SH, DMS, and DMDS) was initially purchased (Ri Gas, Corp., Korea). These primary standards were then used after dilution using a 10 mL gas-tight syringe. To facilitate the calibration of RSC, the system was operated in the forced linear mode with the square root function on. More details of the Reduced Sulfur Compounds analysis are given in Table 2.

### 2.6. Analysis of Ammonia

The colorimetric indophenol blue technique was used to analyze the air samples for their gaseous ammonia content. The indophenol method for determining ammonia in air and aerosol samples is based on the formation of an indophenol blue pigment during the reaction of phenol and hypochlorite in the presence of ammonia. The absorbing reagent (10 mL) was placed in the impinger and the sampling train was assembled in the following manner: inverted funnel, pre-filter (pre-washed Whatman No. 41), impinger, moisture trap (U-tube with silica gel), rotameter and pump. Air samples were passed through at a flow rate of 5L/min. The level of the sampling reagent in the impinger before sampling was marked and it was made up to the mark with water after sampling to compensate for the loss due to evaporation.

### 2.7. Analysis of Trimethylamine

Analysis of trimethylamine was performed with a Solid Phase Microextration (SPME) fiber [1], accompanied with a GC/NPD (Nitrogen Phosphorous Detector). Sixty five micrometer diameter PDMS-divinylbenzene was used as SPME fiber for adsorption of trimethylamine. The SPME adsorption process was performed at a constant temperature with the help of an incubator. The trimethylamine analysis instrument was a GC-NPD (SHIMADU 17A, Japan). The column for GC was crompack volamine (60 m × 0.32 mm × 0.45 μm, Varian). Oven temperature was maintained at 60 °C for 20 min and then increased to 250 °C at a rate of 20 °C/min. It was maintained at 250 °C for 3 min.

Air and hydrogen gas flows to the GC were 80 and 30 mL/min each. The temperature for the NPD was 250 °C and the current was set at 80 pA. Helium (99.999%) was used as the carrier gas. Flow pressure at the column was set at 60 kPa for 20 min and was increased to 135 kPa at a rate of 10 kPa/min. It was maintained at 135 kPa for 5 min. Ninety five ppm of CRM (Certified Reference Material) from the Korean Research Institute of Standards and Science (KRISS) was used as the standard gas for trimethylamine. Dilution for standard gas was performed based on volume ratio with a Tedlar bag (polyvinyl fluoride bag, SKC. Inc, USA).

## 3. Results and Discussion

Table 4 shows the summer time measurement results of the selected odorous compounds at the Wastewater Treatment Plant. At the primary settling basin, generally higher concentrations of the odorous compounds (except hydrogen sulfide) were measured. Ammonia recorded the highest concentration (506 ppb), followed by dimethyl disulfide (207 ppb). Dimethyl disulfide had the biggest concentration fluctuation.

Table 5 shows the measurements of the selected odorous compounds during winter. During winter, relatively higher concentrations of hydrogen sulfide and ammonia were detected at the primary settling basin. In the case of the other odorous compounds, higher concentrations were detected at the aeration basin.

Aneja *et al.* found out that the average flux rate for ammonia from six anaerobic waste water storage and treatment lagoons (primary, secondary and tertiary) was in the range of 40.7 – 120.3 μg/m^2^/min [20]. Our study shows an average flux rate for ammonia in the range of 97 – 870 μg/m^2^/min. Byler *et al.* [21] in their study on odor emission rates from phototropic lagoons estimated the emission rates of hydrogen sulfide to be 6 – 114 μg/m^2^/min. Catalan *et al.* [22] found that the average flux rate from the surfaces of primary and secondary wastewater clarifiers were in the 0.066 – 23.4 μg/m^2^/min range for hydrogen sulfide, 0.066 – 11.4 for methyl mercaptan, 0.00144 – 10.2 μg/m2/min for dimethyl sulfide and 0.0336 – 49.8 μg/m^2^/min for dimethyl disulfide. This is slightly different from the results of our study which reveals average flux rates between the ranges of 0.08 – 22.05 μg/m^2^/min for hydrogen sulfide, 0.08 – 5.84 for methyl mercaptan, 0.41 – 4.32 for dimethyl sulfide and 0.35 – 105.47 μg/m^2^/min for dimethyl disulfide.

The Dynamic Flux Chamber (DFC) gave estimates of emission fluxes of the odorous compounds (μg/m^2^/min). The odorous compounds fluxes were calculated by considering the mass balance of odors in the DFC [20]. The fluxes were estimated by using the following equation (1):

(1) $$J=\frac{V}{A}\left(L\frac{A_{C}}{V}+\frac{Q}{V}\right)C$$

where:

J : Odor compound fluxes expressed as mass per area per time (μg/m^2^/min)
V : Volume of DFC (m^3^)
A : Water surface area covered by DFC (m^2^)
L : The loss rate from the chamber wall per unit area as first order in concentration (m/min)
AC : Surface area of the inner walls of DFC (m^2^)
Q : Flow rate within the DFC (m^3^/min)
C : Concentrations of odor compounds in the DFC (μg/m^3^)

Loss rate is the loss that occurs due to the reaction with the inner surfaces of the chamber. Roelle *et al.* [23] and Aneja *et al.* [24-25] estimated the ammonia sampling loss rate of the DFC to be 0.02760 m/min and 0.01723 m/min respectively. In order to account for possible loss from the chamber system, we used the average loss rate of these two values in our study, assuming that they hold true for our experiment as well, since the same chamber system was used. Table 6 shows the averaged emission flux (μg/m^2^/min) from the WWTP for each selected odorous compound.

**Figure 4.**
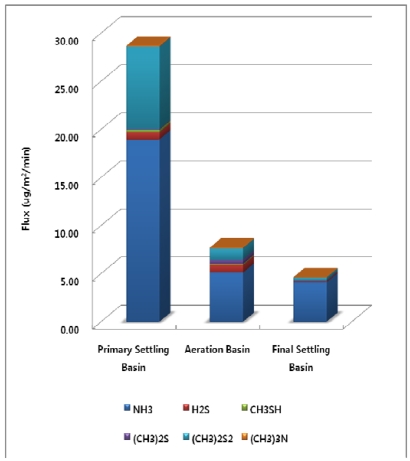
Annual Odorous Compounds Emission Flux from each Treatment Process.

At the WWTP, the surface area of the primary settling basin is 1872 m^2^, the area of the aeration basin is 5,760 m^2^, and the area of the final settling basin is 5,024 m^2^. Surface areas of the treatment processes at the Wastewater Treatment Plant were used for estimation of emission flux Figure 4 illustrates how the annual total odorous compounds emission flux per unit area is highest at the primary settling basin (28.72 μg/m^2^/min). Odorous compounds emission fluxes for the aeration basin and final settling basin were 7.71 and 4.62 μg/m^2^/min each.

However, as the surface areas for the aeration basin (5760 m^2^) and the final settling basin (5024 m^2^) are larger than that of the primary settling basin (1872 m^2^), the total amount of the selected odorous compounds emission for the treatment processes are similar with each other. The total amount of the selected odorous compounds emissions per year for the primary settling basin, aeration basin, and final settling basin were 28.3, 23.3 and 12.2 kg/year respectively.

Table 7 shows the amount of the odorous compounds emitted per treated wastewater ton from each treatment process.

**Figure 5.**
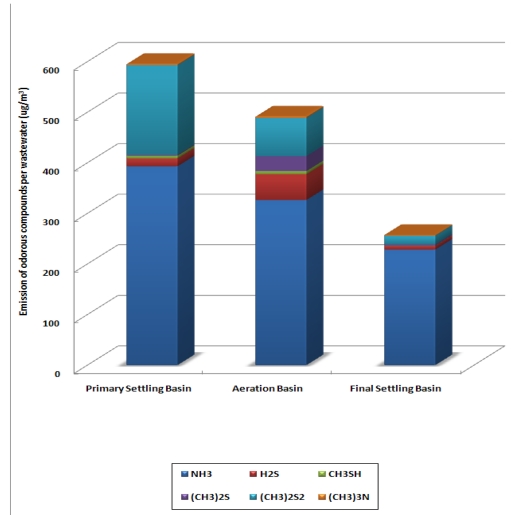
Amount of the Selected Odorous Compounds Emitted per Treated Wastewater (μg/m^3^) from Each Treatment Process.

The total amount of the selected odorous compounds emitted per wastewater cubic meter was 1,334 μg/m^3^ from each treatment processes. From the primary settling basin, 595 μg of odorous compounds were emitted per cubic meter of wastewater and from the aeration basin and the final settling basin, 492 and 257 μg each was emitted. Figure 5 illustrates the amount of annual average odorous compounds per treated wastewater cubic meter (μg/m^3^) for each treatment process.

Table 8 shows the selected odorous compounds' composition flux ratio from each treatment process.

**Figure 6.**
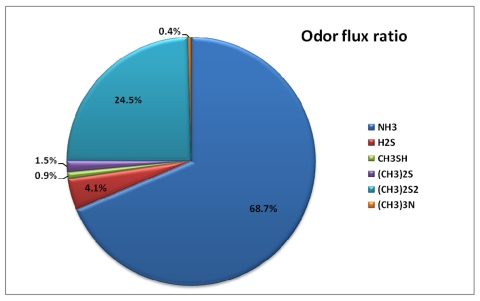
Annual Emission of odorous compounds Flux Composition Ratio and Odor Intensity Contribution Ratio.

Out of all the selected odorous compounds, ammonia occupied the biggest portion. However, the emission flux composition ratio increased from the primary settling basin (66.0%) to the final settling basin (88.9%). To observe the odor intensity contribution ratio from each odorous compound, the measured concentration was divided by its own threshold value. Odor intensity contribution ratios are dramatically different when compared to emission flux composition ratio. Figure 6 and Table 8 show the odor intensity contribution ratio for each odor compound.

Even though the composition ratio for ammonia is dominant at all the treatment processes, the dominant odor intensity contribution was caused by dimethyl disulfide (69.1%). During summer, relatively higher amounts of the selected odorous compounds were emitted compared to that of winter. This may have been caused by higher temperatures during summer.

## 4. Conclusions

Emission characteristics of six odorous compounds from a wastewater treatment plant at Sun-Cheon, Korea were investigated. To evaluate their emission fluxes from the WWTP, a Dynamic Flux Chamber (DFC) was used. The targeted odorous compounds selected were hydrogen sulfide, methyl mercaptan, dimethyl sulfide, dimethyl disulfide, ammonia, and trimethylamine. Higher concentrations of the odorous compounds (except hydrogen sulfide) were detected at the primary settling basin. During winter, relatively higher concentrations of hydrogen sulfide and ammonia were detected at the primary settling basin. In the case of the other odorous compounds, higher concentrations were detected at the aeration basin.

Annual total of selected odorous compound emission flux per unit area of the primary settling basin was 28.72 μg/m^2^/min. Odorous compounds emission fluxes for the aeration basin and the final settling basin were 7.71 and 4.62 μg/m^2^/min each. Total amount of selected odorous compounds emission per year for the primary settling basin, aeration basin, and final settling basin were 28.3, 23.3 and 12.2 kg/year respectively. During summer, relatively higher amounts of the odorous compounds were emitted compared to winter. This may have been caused by higher temperatures during summer.

An average flux rate for ammonia was in the range of 97 – 870 μg/m^2^/min. In the case of hydrogen sulfide, it was measured between the range of 0.08 – 22.05 μg/m^2^/min and it was 0.08 – 5.84 for methyl mercaptan, 0.41 – 4.32 for dimethyl sulfide, 0.35 – 105.47 μg/m^2^/min for dimethyl disulfide each.

Five hundred ninety five μg of selected odorous compounds were emitted per treated wastewater cubic meter from the primary settling basin, while from the aeration basin and final settling basin, 492 and 257 μg/m^3^ were emitted each. In the case of ammonia, the emission flux composition ratio increased from the primary settling basin (66.0%) to the final settling basin (88.9%). Even though the composition ratio for ammonia is dominant at all the treatment processes, the dominant odor intensity contribution was caused by dimethyl disulfide (69.1%).

## Figures and Tables

**Table 1.** Selected Odorous Compounds from Wastewater Treatment Plant and their Corresponding Odor Threshold values.

**Compound**	**Odor Threshold (ppm)**	**Characteristic Odor**
Hydrogen sulfide (H_2_S)	0.0005^a^	Rotten eggs
Methyl mercaptan (CH_3_SH)	0.0016^b^	Decayed cabbage
Dimethyl sulfide ((CH_3_)_2_S)	0.001^a^	Decayed vegetables
Dimethyl disulfide ((CH_3_)_2_S_2_)	0.003^c^	Vegetable sulfide
Ammonia (NH_3_)	5.2^b^	Pungent, irritating
Triemethylamine ((CH_3_)_3_N)	0.0004^a^	Ammonical, fishy

a WEF Manual of Practice No. 22 ASCE Manuals and Reports on Engineering Practice No. 82 [15].

b Guide to Field Storage of Biosolids – Odor Characterization, Assessment and Sampling [16].

c Annual Reports of 1990 – Japan Environment Sanitation Center [17].

**Table 2.** Temperature and Pressure of Ambient Air, DFC and Sewage Surface during Sampling.

**Sampling date and points**			**Temperature (°C)**			**Ambient pressure(mmHg)**
Date(season)		Sampling Points	Ambient	DFC	Sewage	
Summer	A.M	Primary settling basin	31.5	29.3	22.0	751.5
		Aeration basin	31.0	26.8	27.9	
		Final settling basin	29.5	27.9	23.0	
	P.M	Primary settling basin	31.9	30.4	22.9	
		Aeration basin	32.4	28.6	26.1	
		Final settling basin	31.4	32.1	23.5	
Winter	A.M	Primary settling basin	11.2	12.6	14.1	756.5
		Aeration basin	9.8	16.4	11.6	
		Final settling basin	10.1	12.9	13.2	
	P.M	Primary settling basin	9.1	18.2	14.0	
		Aeration basin	10.6	11.7	13.0	
		Final settling basin	10.5	14.6	12.7	
Winter	A.M	Primary settling basin	9.5	9.5	12.2	754.6
		Aeration basin	7.8	8.9	11.5	
		Final settling basin	8.4	14.7	11.7	
	P.M	Primary settling basin	9.4	23.3	12.1	
		Aeration basin	9.2	18.7	11.7	
		Final settling basin	9.4	20.4	11.4	

**Table 3.** Operational Condition of TDU-GC/PFPD for Reduced Sulfur Compounds.

**TDU**		**GC/PFPD**		
Cold trap packing	Carbopack B+Silica Gel	Carrier gas		N_2_
		flow	Column	20 psi
			Air (1)	10 mL/min
			Air (2)	10 mL/min
			H_2_	11.5 mL/min
Adsorption flow	5∼10 mL/min	GC Column		BP-1 (60 m × 0.32 mm, 5.0 um)
Cold trap high temp.	300 °C			
Cold trap low temp.	-15 °C	Oven Temp.		40 °C (10min) − 5 °C/min
Hold time	5.0 min			
Outlet split	5.0 mL/min(5:1 split ratio)			- 200 °C (5min)
Flow path temp.	80 °C			

**Table 4.** Measurements of the Selected Odorous Compounds during Summer Time.

**Treatment Process**	**Unit: ppb**	**NH_3_**	**H_2_S**	**CH_3_SH**	**(CH_3_)_2_S**	**(CH_3_)_2_S_2_**	**(CH_3_)_3_N**
Primary settling basin	Morning	506	23.86	7.34	No detection	207.90	2.84
	Afternoon	340	20.23	4.34	No detection	3.03	1.33
	Mean	423	22.05	5.84		105.47	2.09
	SD	117	2.57	2.12		144.86	1.07
Aeration basin	Morning	120	26.79	No detection	No detection	2.20	No detection
	Afternoon	270	26.62	No detection	No detection	1.75	No detection
	Mean	195	26.71			1.98	
	SD	106	0.12			0.32	
Final settling basin	Morning	181	4.35	No detection	No detection	3.56	No detection
	Afternoon						
	Mean	181	4.35			3.56	
	SD						
Low Detection Limit		7.50	0.14	0.17	0.15	0.17	0.22

**Table 5.** Measurements of the Selected Odorous Compounds during Winter Time.

**Treatment Process**	**Unit: ppb**		**NH_3_**	**H_2_S**	**CH_3_SH**	**(CH_3_)_2_S**	**(CH_3_)_2_S_2_**	**(CH_3_)_3_N**
Primary settling basin	1^st^ Measur.	Morning	780	1.68	0.35	1.11	6.12	No detection
		Afternoon	783	9.85	0.43	0.80	2.44	0.17
	2^nd^ Measur.	Morning	1,047	0.08	0.01	0.74	0.05	No detection
		Afternoon	944	2.27	0.05	0.53	0.15	No detection
	Mean		870	3.47	0.21	0.80	2.19	0.04
	SD		153	4.35	0.21	0.24	2.84	
Aeration basin	1^st^ Measur.	Morning	105	0.13	1.09	2.87	23.51	0.13
		Afternoon	275	0.11	0.86	3.61	23.48	
	2^nd^ Measur.	Morning	49	0.03	1.74	7.16	2.71	
		Afternoon	170	0.05	0.87	3.64	1.32	0.72
	Mean		150	0.08	1.14	4.32	12.75	0.43
	SD		97	0.05	0.41	1.93	12.41	0.41
Final settling basin	1^st^ Measur.	Morning	164	0.06	0.04	0.33	0.54	0.32
		Afternoon	163	0.07	0.03	0.39	0.53	0.17
	2^nd^ Measur.	Morning	52	0.15	0.14	0.42	0.11	0.16
		Afternoon	10	0.02	0.09	0.51	0.20	0.23
	Mean		97	0.08	0.08	0.41	0.35	0.22
	SD		79	0.05	0.05	0.07	0.22	0.07

**Table 6.** Odorous Compounds Emission Flux (μg/m^2^/min) from the Wastewater Treatment Plant.

**Season**	**Treatment Process**	**Each Odorous Compound Flux(μg/m^2^/min)**					
		**NH_3_**	**H_2_S**	**CH_3_SH**	**(CH_3_)_2_S**	**(CH_3_)_2_S_2_**	**(CH_3_)_3_N**
Annual Average	Primary Settling Basin	18.96	0.75	0.25	0.09	8.56	0.12
	Aeration Basin	5.12	0.80	0.10	0.47	1.18	0.04
	Final Settling Basin	4.11	0.13	0.01	0.04	0.31	0.02
Summer	Primary Settling Basin	12.41	1.30	0.49	Not detected	16.76	0.21
	Aeration Basin	5.79	1.59	Not detected	Not detected	0.32	Not detected
	Final Settling Basin	5.34	0.26	Not detected	Not detected	0.57	Not detected
Winter	Primary Settling Basin	25.52	0.20	0.02	0.09	0.35	0.02
	Aeration Basin	4.45	0.01	0.10	0.47	2.05	0.04
	Final Settling Basin	2.87	0.00	0.01	0.04	0.06	0.02

**Table 7.** Amount of Emitted Odorous Compounds per Treated Wastewater (μg/m^3^).

**Treatment Process**	**Odor Compound emission per treated wastewater (μg/ m^3^)**						**Total Emi. (μg/m^3^)**
	**NH_3_**	**H_2_S**	**CH_3_SH**	**(CH_3_)_2_S**	**(CH_3_)_2_S_2_**	**(CH_3_)_3_N**	

Primary Settling Basin	393	16	5	2	177	2	595

Aeration Basin	327	51	6	30	76	3	492

Final Settling Basin	229	7	0	2	17	1	257

Total	948	74	12	34	270	6	1344

**Table 8.** Odorous Compounds Emission Flux Composition Ratio and their Intensity Contribution Ratio with respect to the Six Selected Odorous Compounds in WWTP.

**Ratio**	**Treatment Process**	**Each Odorous Compound Ratio (%)**					
		**NH_3_**	**H_2_S**	**CH_3_SH**	**(CH_3_)_2_S**	**(CH_3_)_2_S_2_**	**(CH_3_)_3_N**

Emission Flux Ratio	Primary Settling Basin	66.0%	2.6%	0.9%	0.3%	29.8%	0.4%

	Aeration Basin	66.4%	10.3%	1.2%	6.1%	15.4%	0.6%

	Final Settling Basin	88.9%	2.8%	0.1%	1.0%	6.8%	0.5%

	Total	68.7%	4.1%	0.9%	1.5%	24.5%	0.4%

Odor Intensity Ratio	Primary Settling Basin	0.0%	4.3%	4.2%	1.3%	88.4%	1.7%

	Aeration Basin	0.0%	17.2%	6.1%	27.8%	46.4%	2.5%

	Final Settling Basin	0.1%	14.6%	2.0%	13.5%	63.3%	6.5%

	**Total**	**0.0%**	**10.4%**	**4.9%**	**13.2%**	**69.1%**	**2.4%**
